# Influence of ultrasound-assisted extraction of bioactives from garlic (*Allium sativum*) sprouts using response surface methodology

**DOI:** 10.1016/j.ultsonch.2025.107286

**Published:** 2025-02-25

**Authors:** Waseem Khalid, Imed E. Benmebarek, Kanza Saeed, Hyrije Koraqi, Andres Moreno, Robert Mugabi, Gulzar Ahmad Nayik, Tuba Esatbeyoglu

**Affiliations:** a Department of Organic Chemistry, Faculty of Chemical Sciences and Technologies, University of Castilla La Mancha, 13071 Ciudad Real, Spain; b Department of Molecular Food Chemistry and Food Development, Institute of Food and One Health, Gottfried Wilhelm Leibniz University Hannover, Hannover, Germany; c Faculty of Food Technology & Nutrition Sciences, Lahore University of Biological and Applied Sciences, Lahore 53400, Pakistan; d Faculty of Food Science and Biotechnology, UBT-Higher Education Institution, Pristina, Kosovo; e Department of Food Technology and Nutrition, Makerere University, Kampala, Uganda; f Marwadi University Research Centre, Department of Microbiology, Marwadi University, Rajkot, Gujarat 360003, India

**Keywords:** Polyphenol, Non-thermal treatment, Nutritional composition, Microbial load, Color

## Abstract

Garlic sprouts have nutritional and culinary significances due to good source of chemical compounds. The present study aimed to determine the optimized condition for maximum extraction of polyphenols from garlic sprouts through RSM (response surface methodology). However, the proximate, color and microbial analysis were performed on the freeze-dried garlic sprouts. The influences of extraction time (3–9 min), amplitude (50–90 %), ethanol concentration (25–75 %) and solid–liquid ratio (20–40 g/mL) on the extraction yield and total phenolic content (TPC) were investigated. The highest yield was observed on time (9 min), amplitude (70 %), ethanol (25 %) and solid–liquid ratio (30 g/mL) whereas the maximum TPC were measured on time (3 min), amplitude (70 %), ethanol (75 %) and solid–liquid ratio (30 g/mL). The optimal extraction conditions were 5.20 min, 61.1 % ethanol, 71.4 % amplitude and 30.6  g/mL solid–liquid ratio. However, the estimated results of the study are in accordance with the experimental outputs. In addition, chromatographic measurement showed the characterization of polyphenols in garlic sprout extract. Garlic can be fruitful in pharmaceutical and food industries due to a good source of bioactive compounds.

## 1 Introduction

Food technologists, scientists, and industrialists are considering finding new natural food sources with the increased worldwide population. It would be suitable if these food sources are cheaper and easily available. Nowadays, many people from different countries are interested in developing or creating new and easy food sources [1]. However, over time the trend of kitchen gardens has been increasing. Also, people grow sprouts in the house or kitchen and sometimes when we keep vegetables (onion, garlic, potatoe, etc.) for a long time, some nano-green part is visible, called sprouts [2]. Sprouts are nano-green that are visible when seeds start to grow. Sprouts are are from different sources including vegetable sprouts, cereal sprouts, grain sprouts, and lentil [3].

Garlic belonged to the onion family Alliaceae, and botanically called *Allium sativum.* Garlic contain various phytochemicals [4], [5]. Garlic has been regarded as a potential therapeutic and medicinal plant throughout most of human civic history [6], [7]. Garlic sprouts are composed of different nutrients such as amino acids, polyunsaturated fatty acids, and other micronutrients. Moreover, garlic sprouts are also composed of different types of bioactive compounds that can be extracted using different technologies [4], [8]. Garlic bulbs and leaves are used as aromatic spices that enhance food flavor during cooking [9]. The different parts of garlic contained more than 130 compounds including, organic sulfur compounds, vitamins, protein, amino acids, polysaccharides, flavonoids, volatiles, steroidal saponins and cerebrosides [10]. The pharmacological potential of *A. sativum* is primarily focused on immune regulation, treatment of cardiovascular, anti-tumor potential and pathogenic microorganism resistance [11].

There are two types of extraction methods including conventional and novel. Now, researchers prefer to use novel extraction methods due to better extraction yields as compared to traditional methods [12]. The better yield may be due to control conditions. However, the ultrasound-assisted extraction (UAE) is a novel method that is used to extract bioactive compounds from different types of plant foods. UAE technique has the potential to induce the phenomenon of cavitation through compression and expansion after penetrating the medium [13]. Moreover, the cavitation facilitates maximizing the solvent entry within the cell matrix, and also UAE is cost-effective and economical [14], [15]. The technique has simpler applications and minimum inclusion of dispersed substances that lead to exceptionally pronounced proximity among the determined quantity and its reference value [16]. In addition, UAE improves energy cost rationalization [17], reduces the processing time, and diminishes the possibility of thermal degradation of target compounds [18].

The optimization technique is a tool that enhances the process efficiency to attain better process outputs by systematically adjusting the parameters and variables. The primary objective of optimization is to maximize the benefits and to minimize the losses [19]. The most common approach to evaluate the output of process optimization is response surface methodology (RSM) [20]. RSM is a collective mathematical and statistical approach based on the influence and impact of several different variables on the desired responses [21]. The optimal extraction parameters and conditions can significantly improve the phytochemical and antioxidant potential of *Allium sativum* sprouts. However, further research needs to be done on the bioavailability of the bioactive components and effective formulation development. To expand its potential application on commercial scale, safety and efficacy trials can be established. The current study was focused on analyzing the impact of different conditions (ethanol concentration, time, amplitude, and solid-liquid ratio) on garlic sprout yield and TPC using RSM. HPLC was performed to confirm the phenolic compounds in garlic sprouts extract.

## 2 Material and methods

### 2.1 Raw material

Red garlic bulbs were purchased from Mercadona, Cuidad Real, Spain. The skin was removed and washed with water. Then, garlic bulbs were placed in a plastic bottle at 25 °C for sprouting. After 10 days, garlic sprouts were harvested and put into a freeze-dryer for 48 h. However, freeze-dried garlic sprouts were ground into fine powder using a Lab mill (PULVERISETTE 14 rotor mill, FRITSCH, Idar-Oberstein, Germany) and stored at 4 °C for further analysis. Freeze-dried garlic sprouts were used for extraction.

### 2.2 Chemicals

Gallic acid (97.5–102.5 % titration), Folin–Ciocalteu, sodium carbonate, and ethanol were purchased from Sigma–Aldrich (Steinheim, Germany). Deionized water was used throughout the study.

### 2.3 Proximate and microbial analysis

Moisture, protein, fat, and ash content of freeze-dried garlic sprouts were measured using the method of AOAC [22]. To determine total bacterial count (TBC) of garlic sprout powder, the sample was added to normal saline solution. Sample solution was then homogenized, and 1 mL of diluted sample was placed in a sterile petri dish, followed by addition of plate count agar to the Petri dish. For even mixing of the culture media, the plates were rotated 5 times both clockwise and anticlockwise. Petri dishes were then incubated at 25 °C for the time period of 48 h. The bacterial count was recorded as colony forming units per gram (CFU/g). To determine yeast and mold count, 1 g of the garlic sprout powder was diluted 10-fold with saline solution and homogenized. Serial dilutions were made, and appropriate dilutions were plated on sterilized yeast extract glucose chloramphenicol agar and autoclaved at 121 °C for 15 min. The results were recorded as colony forming units per gram (CFU/g) and all tests were performed in triplicates.

### 2.4 Colorimeter measurements

The color (L* (lightness; degree of brightness or darkness), a* (greenness to redness), b* (blueness to yellowness), c* (chroma), and h (hue angle) of the garlic sprout powder was assessed using a colorimeter (CM5; Konia Minolta, Langenhagen, Germany). A quartz (glass) cuvette filled with garlic sprout powder was introduced into the sample compartment of the cell transmittance. The compartment was closed, and the analysis was performed. The color values were monitored by a computerized system using Spectra Magic Software (Konica Minolta Sensing, Inc.). All measurements were performed in the wavelength range of 360–740 nm. An instrument was set up for an observer angle of 10° and an illuminant of D65.

### 2.5 Ultrasound assisted extraction and extraction yield of garlic sprouts

The sample was placed into 250 mL beaker then solvent was added. The ultrasound-assisted extraction (UP200S, Hielscher, Teltow, Germany) was performed for the extraction purpose. The ultrasound was applied after adjusting the probe up to 4 cm inside the sample. Overheating of the sample during the ultrasound treatment was prevented by covering the beaker with ice. Ultrasound treated samples were centrifuged at 8,000 rpm for 18 min at room temperature and were filtered through filter paper. To determine the yield, *Allium sativum* sprout extract was freeze-dried. Then, the extract was stored at 4 °C for further analysis. The extraction yield (%) was measured by comparing the dry weight of the extract with the initial weight of garlic sprouts using Eq 1.

(1) $$Extraction\;yield\;\left(\%\right)=\frac{\mathrm{Weight}\;\mathrm{of}\;\mathrm{dried}\;\mathrm{sprout}\;\mathrm{extract}}{\mathrm{Weight}\;\mathrm{of}\;\mathrm{the}\;\mathrm{initial}\;\mathrm{dried}\;\mathrm{sample}}X100$$

### 2.6 Total phenolic content (TPC)

The total phenolic content of garlic sprout extract was measured using the respected method of Singleton & Rossi. [23] with few modifications. The 0.5 mL sprout extract was mixed with 2.5 mL of Folin. After that, 2 mL of NaCO_3_ (7.5 %) was added and kept for incubation (1 h) at room temperature. Then, the absorbance of the samples was measured at a wavelength of 750 nm. Total phenolic content was expressed as mg gallic acid equivalent (GAE)/g.

### 2.7 Experimental design

Box-Behnken Design of Response surface methodology with 28 runs were performed. Independent variables of the trial results were time (3–9 min), amplitude (50–90 %), ethanol concentration (solvent) (25–75 %) and solid-liquid ratio (20–40 mL/g) with three levels (−1, 0, +1) were used to evaluate the optimum combinations regarding two responses (Table 1). Design expert version 13 (Stat-Ease, Minneapolis, MN, the United States of America) was used for response surface methodology.

**Table 1:** Independent variables and levels of the Box-Behnken Design.

Independent Variable	Symbol		Levels	
		−1	0	+1
Time (min)	A	3	6	9
Amplitude (%)	B	50	70	90
Ethanol concentration or solvent (%)	C	25	50	75
Solid-liquid ratio (g/mL)	D	20	30	40

### 2.8 Characterization of phenolic compounds

The phenolic compounds in garlic sprouts extract was investigated through optimized process using high performance liquid chromatography equipped with diode array detector for the purpose of detection of multi-wavelength. A constant column temperature of 25 °C was maintained. The garlic sprout extract was injected in the column for separation. However, different polyphenols of garlic sprout extract was conducted at 280 nm. The phenolic compounds were identified on the basis of retention time by comparing with the standards

### 2.9 Statistical design

In the current study, Box-Behnken Design was used to determine the impact of ethanol concentration, time, amplitude, solid–liquid ratio across 28 experiments with 4 replicates at central point. Model's level of significance was analyzed using ANOVA to establish and describe relationship between responses and variables.

## 3 Results and discussion

### 3.1 Proximate composition

Garlic sprouts are a good source of phytochemicals. Garlic sprouts contain 8.25 ± 0.15 % moisture, 39.76 ± 1.35 % crude protein, 2.03 ± 0.11 % crude fat and 8.25 ± 0.21 % crude ash content as shown in Table 2. Zakarova et al. [8] proved that garlic sprouts are an important source of phytochemicals that can improve health. Another study related to our results focused on the nutritional composition of garlic leaves. The results showed that garlic leaves contain different chemical compounds including protein, fat, carbohydrates and ash [4]. Similarly, Skoczylas et al. [24] performed a study on garlic leaves. The proximate analysis showed that young garlic leaves are composed of more nutritional compounds compared to bulbs.

**Table 2:** Proximate results, microbial load and color measurement of freeze-dried garlic powder.

**Parameter**	**Garlic sprout**	**Mean ± SD**
**Proximate** (%)	Moisture	8.25 ± 0.15
	Crude protein	39.78 ± 1.35
	Crude Fat	2.03 ± 0.11
	Crude Ash	8.25 ± 0.21
**Microbial** (log_10_CFU/g)	TBC	4.57 ± 0.02
	Yeast/Mold	<10
**Colorimeter measurement**	L*	59.37 ± 0.04
	a*	−9.17 ± 0.01
	b*	28.49 ± 0.02
	c*	29.93 ± 0.02
	h	107.84 ± 0.01

### 3.2 Microbial analysis of garlic sprout powder

The microbial analysis represents the total number of colonies of bacteria, yeast and mold. The results of total microbial count of garlic sprout powder are exhibited in Table 2. Total bacterial count is 4.567 ± 0.016 log_10_CFU/g and total yeast and mold count is < 10 log_10_CFU/g. Lemma et al. [25] showed that garlic bulb and leek leaf oil demonstrated considerable biological activities as antioxidant and antimicrobial potentials. However, garlic bulb oil demonstrated better antifungal potential. *Allium* plants (garlic, onion and leek) are rich in hydrophobic antimicrobial compounds like allicin, vinyldithiins, ajoenes and diallyl polysulfides. Allicin is a sulfur containing bioactive compound with strong antimicrobial potential against yeasts, molds and both Gram-positive and Gram-negative bacteria. Allicin also inhibit formation of bacterial biofilms, which are the cause of bacterial resistance against antibiotic treatment of infectious diseases, through quorum sensing regulation [26]. Due to strong antimicrobial potential of bioactive components of garlic sprouts, total bacterial, and yeast and mold count of garlic sprout powder in current study is low. Oyawoye et al. [27] studied the antimicrobial potential of garlic against multi-drug-resistant bacteria and the results proved the effectiveness of bioactive components of garlic. The outcomes of study demonstrated antimicrobial potential of *A. sativum* against *Staphylococcus aureus*. Findings of the current study well corroborate with these reports regarding antimicrobial potential of garlic against microorganisms.

### 3.3 Color of garlic sprout powder

Consumers assess the food quality and acceptability based on its color parameters. In this study CIE color system was used to determine the color of garlic sprout powder. The analyzed values of garlic sprout powder were L*(59.37 ± 0.04), a* (−9.17 ± 0.01) and b* (28.49 ± 0.02), chroma (29.93 ± 0.02) and hue angle (107.84 ± 0.01) as shown in Table 2. Prakash and Prasad [28] conducted a study on quality assessment of different garlic varieties. The results exhibited that the color variation among the varieties is due to pigmenting compounds like anthocyanins. According to a study conducted by Chan et al. [29] pre-treatments like freezing and high-pressure induce porosity, structural deformations and enzymatic browning. Due to structural damage, cell membrane bounded polyphenols and oxidative enzymes like polyphenol oxidase are released, resulting in enzymatic browning reactions in garlic. During storage the color of garlic darkens under high temperature and humid storage environment, fructans cleaves into fructose and glucose and react with amino acids, resulting in formation of melanoidins which darken the garlic [30].

### 3.4 System model of yield and TPC

The impact of four extraction parameters (time, amplitude, ethanol concentration, solid–liquid ratio) was determined on extraction yield and total phenolic content. The responses (yield and TPC) of 28 run independent variables are summarized in Table 3.

**Table 3:** Box-Behnken experimental design and its responses for yield and total phenolic content of garlic sprouts.

Run	Factor A: Time (min)	Factor B: Amplitude (%)	Factor C: Ethanol concentration (%)	Factor D: Solid-liquid ratio (g/mL)	Response: Yield (%)	Response: TPC (mg GAE/g)
1	6	70	75	40	41.0	5.40
2	6	90	25	30	52.0	7.00
3	9	70	50	20	49.0	3.24
4	6	50	25	30	52.0	6.47
5	3	50	50	30	45.0	7.50
6	9	70	50	40	42.4	3.85
7	6	50	50	20	52.0	5.20
8	6	70	50	30	40.0	7.45
9	3	70	50	40	41.5	3.75
10	6	70	50	30	40.0	7.60
11	3	70	75	30	45.5	8.20
12	6	70	50	30	41.0	7.10
13	6	70	25	40	49.0	3.20
14	6	70	75	20	45.0	4.38
15	9	50	50	30	48.3	5.30
16	9	90	50	30	48.0	6.73
17	3	70	50	20	44.0	6.01
18	6	70	25	20	52.0	5.93
19	9	70	75	30	41.0	6.70
20	6	90	50	20	45.5	5.50
21	9	70	25	30	53.6	5.50
22	6	90	50	40	45.8	4.27
23	3	90	50	30	45.2	7.80
24	6	70	50	30	39.0	7.20
25	6	90	75	30	44.0	7.50
26	6	50	75	30	46.0	7.45
27	6	50	50	40	40.5	4.24
28	3	70	25	30	47.6	7.08

Analysis of variance was used to calculate the regression coefficient. For each parameter, results were expressed as statistical significance (*p* > 0.05) and statistical non-significance (*p* < 0.05). Model adequacy was indicated and expressed in the form of coefficients of multiple determination. Therefore, the regression model equation can predict the relationship between independent factors ultrasound time (min) (A), amplitude (%) (B), ethanol concentration (%) (C), and solid–liquid ratio (g/mL) (D) for yield and TPC are summarized in eq. (2) and eq. (3) respectively.

(2) $$\begin{array}{c} Yield\left(\%\right)=40.00+1.12\times A-0.2750\times B-3.64\\ \times C-2.28\times D-0.1250\times AB-2.63\times AC-1.03\\ \times AD-0.5000\times BC+2.95\times BD-0.2500\times CD\\ +2.39\times A^{2}+4.04\times B^{2}+4.59\times C^{2}+1.97\times D^{2} \end{array}$$

(3) $$\begin{array}{c} TPC\left(\mathrm{mgGAE}/g\right)=7.34-0.7517\times A+0.2200\times B\\ +0.3708\times C-0.4625\times D+0.2825\times AB+0.0200\\ \times AC+0.7175\times AD-0.1200\times BC-0.0675\times BD\\ +0.9375\times CD-0.4696\times A^{2}-0.0571\times B^{2}-0.0758\times C^{2}-2.56\times D^{2} \end{array}$$

The R2, adjusted R2 and predicted R2 values of yield were 0.983, 0.964, and 0.916 respectively. However, R2, adjusted R2 and predicted R2 values of TPC were 0.984, 0.966 and 0.915 respectively. These values indicated that the model of second order polynomial exhibited a close approximation to the results obtained through experimentation as shown in Table 4. Findings of analysis of variance (ANOVA) showed that (*p* < 0.05) regression model was significant for both yield and total phenolic content. Moreover, the outcomes of lack fit were non-significant (*p* > 0.05) for yield and total phenolic content. The results proved that regression equation is a reliable source predictor for the investigated responses in experimental design (Table 4).

**Table 4:** Analysis of variance (ANOVA) of yield and total phenolic content of garlic sprout extract.

**Source**	**Yield(%)**					**TPC(mg GAE/g)**				
	**Sum of Square**	**df**	**Mean Square**	**F-value**	***p*-value**	**Sum of Square**	**df**	**Mean Square**	**F-value**	***p*-value**
**Model**	488.39	14	34.88	52.52	<0.0001	60.24	14	4.30	55.57	<0.0001
**A-time**	15.19	1	15.19	22.86	0.0004	6.78	1	6.78	87.55	<0.0001
**B-amplitude**	0.9075	1	0.9075	1.37	0.2634	0.5808	1	0.5808	7.50	0.0169
**C-solvent**	159.14	1	159.14	239.59	<0.0001	1.65	1	1.65	21.31	0.0005
**D-solid-liquidratio**	62.11	1	62.11	93.50	<0.0001	2.57	1	2.57	33.15	<0.0001
										
**AB**	0.0625	1	0.0625	0.0941	0.7639	0.3192	1	0.3192	4.12	0.0633
**AC**	0.0625	1	0.0625	41.50	<0.0001	0.0016	1	0.0016	0.0207	0.8879
**AD**	4.20	1	4.20	6.33	0.0258	2.06	1	2.06	26.59	0.0002
**BC**	1.0000	1	1.0000	1.15	0.2416	0.0576	1	0.0576	0.7438	0.4041
**BD**	34.81	1	34.81	52.41	<0.0001	0.0182	1	0.0182	0.2353	0.6237
**CD**	0.2500	1	0.2500	0.3764	0.5501	3.25	1	3.25	45.40	<0.0001
**A^2^**	34.32	1	34.32	51.67	<0.0001	1.32	1	1.32	17.08	0.0012
**B^2^**	98.01	1	98.01	147.55	<0.0001	0.0196	1	0.0196	0.2525	0.6237
**C^2^**	126.50	1	126.50	190.45	<0.0001	0.0345	1	0.0345	0.4456	0.5161
**D^2^**	23.21	1	23.21	34.94	<0.0001	39.19	1	39.19	506.12	<0.0001
**Residual**	8.63	13	0.6642			1.01	13	0.0774		
**Lack of Fit**	6.63	10	0.6635	0.9952	0.5696	0.8498	10	0.0850	1.63	0.3794
**Pure Error**	2.00	3	0.6667			0.1569	3	0.0523		
**Cor Total**	497.02	27				61.25	27			
**R^2^**	0.983					0.984				
**Adjusted R^2^**	0.964					0.966				
**Predicted R^2^**	0.916					0.915				
**Adequacy Precision**	24.1					24.30				

Loghmanifar et al. [31] applied response surface methodology to optimize ultrasound-assisted for the extraction of garlic. The results revealed that RSM effectively optimized the process parameters. Among variables sonication time had the greatest impact on total phenolic content and extraction efficiency, that accelerates the process and significantly improve the extract quality of its antioxidant potential and heat sensitive biologically active components. Similarly, Arteaga-Crespo et al. [32] worked on ultrasonic extraction of total phenolic content using response surface methodology. Experimental finding proved that point distribution confirmed the suitability of model to cover the data of entire analyzed intervals.. The results also validated the similarity of expected and experimental findings.

### 3.5 Effect of ultrasound treatment on yield and total phenolic content

**Fig. 1:**
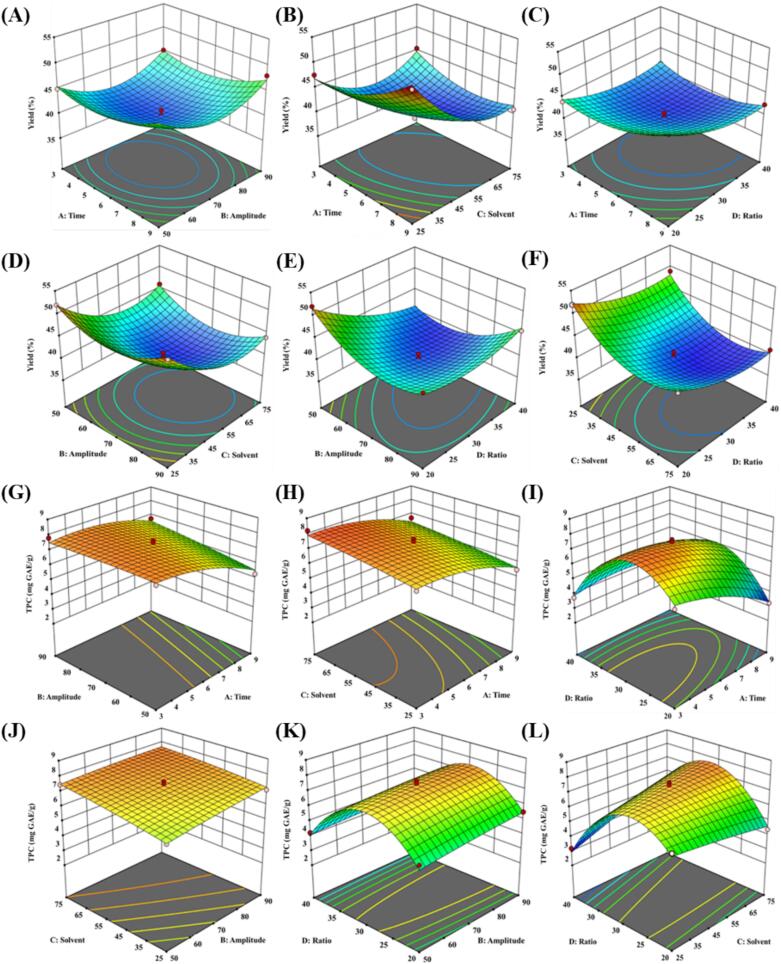
Effect of ultrasound time, amplitude, solvent concentration, solid-liquid ratio on extraction yield (A, B, C, D, E, F) and total phenolic content (G, H, I, J, K, L) of garlic sprout extract (A: time, B: amplitude, C: solvent and D: ratio). Here, the effect of different conditions on yield; A (time and amplitude), B (time and solvent), C (time and ratio), D (amplitude and solvent), E (amplitude and ratio) and F (solvent and ratio). Effect of different conditions on TPC G (amplitude and time), H (solvent and time), I (ratio and time), J (solvent and amplitude), K (ratio and amplitude) and L (ratio and solvent).

The impact of different parameters on yield and total phenolic content is exhibited in Fig. 1 and their significance was calculated using response surface methodology. According to Fig. 1 three-dimensional plot of response surface exhibit interaction between process parameters and yield and total phenolic content of sprout extract. According to the mean values of extraction yield, highest value was attained at time (9 min), amplitude (70 %), ethanol (25 %) and solid–liquid ratio (30 g/mL). According to the mean values of total phenolic content, highest outcome was attained at time (3 min), amplitude (70 %), ethanol (75 %) and solid–liquid ratio (30 g/mL).

The data analysis of variance exhibits a significant effect of process parameters in linear terms i.e., A-time, B-amplitude, C-solvent (ethanol concentration) and D-solid-liquid ratio. In terms of interaction a positive effect was observed in time- ratio and solvent-ratio while opposite effect on interaction was observed in case of time–amplitude, time-solvent, amplitude-solvent and amplitude- ratio.

The increase in treatment time and amplitude positively impacted the total phenolic content yield. Higher temperatures and ultrasonication amplitude soften the cell wall inducing cellular disruption. The results increased in cavitation which gives better yield. In the results of interaction between treatment time and ethanol concentration, an initial increase in the yield of total phenolic content was observed. These results were declined subsequently by an increase in ethanol concentration due to cell wall disintegration, as the solvent acts as a swelling agent and breaks up the plant cell wall. In addition, polyphenols are dissolved in ethanol that is disrupting the solvent solid surface contact area. The yield of total phenolic content dropped after certain limit increase in ethanol concentration, which in turn increased solvent dielectric constant.

Che Sulaiman et al. [33] studied the effects of different extraction process parameter i.e., time, temperatureand solvent ratio on the extraction yield of *Clinacanthus nutans*. The results exhibited that the maximum value was attained between temperature range of 80 °C and water: ethanol ratio of (70:30), extraction time of 120 min. At high temperature, plant tissues soften due to weakening of interactive forces among the molecules of cell membranes, and result in release the phenolic compounds and improving the extraction yield [34]. Similarly, higher the interaction of solvent with plant material results migration of phenolic components into the solvent [35]. In solvent system, water penetrate the plant matrix resulting in swelling of plant material that can contribute to increase yield [36]. However, similar trends were observed by Shekhar et al. [4] for ultrasound extraction of bioactive compounds from garlic leaves. *Allium sativum* is used for medicinal purpose due to its strong antioxidant potential. The antioxidant properties of plants are responsible for anti-cancer, antimicrobial and immune enhancing activity [37], [38]. During the sprouting process, certain biological mechanisms escalate or decrease the antioxidant potential. However, it is a natural process of seed germination that can improve seed nutritive value [39]. Previous study proved that total phenolic content and total flavonoid content in plant species are improved after sprouting. Yang et al. [40] observed changes in phenolic content of garlic by ethanol extract. Majid et al. [41] proved that sprouting can increase phytochemicals. However, outcomes showed that onion sprouting contained more quantity of total phenol content, total flavonoid content, antioxidant activity, anthocyanin content.

### 3.6 Characterization of phenolic compounds by HPLC-DAD

**Fig. 2:**
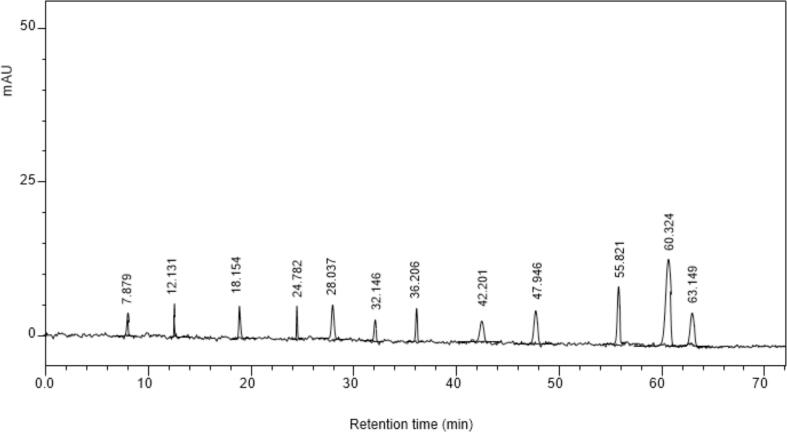
HPLC chromatogram of bioactive compunds from garlic sprouts at 280 nm.

The HPLC chromatogram is shown in Fig. 2. The HPLC results revealed the presence of different polyphenols in garlic sprout extract. The polyphenols were identified by comparing the retention time (Rt) of pure standards of polyphenols attained under the same experimental conditions. The phenolic compounds in garlic sprout extracts identified through chromatographic analysis are: gallic acid at Rt 7.879 min, 3,4-dihydroxybenzoic acid at Rt 12.131 min, chlorogenic acid at Rt 18.154 min, catechin at Rt 24.782 min, syringic acid at Rt 28.037 min, *p*-coumaric acid at Rt 32.146 min, rutin at Rt 36.206 min, ellagic acid at Rt 42.201 min, benzoic acid at Rt 47.996 min, hesperidin at Rt 55.821 min and quercetin at Rt 63.149 min. Highest concentration of 3,4-dihydroxybenzoic acid was observed (3,4-Dihydroxybenzoic acid), followed by rutin (2.447 mg/100 g), catechin (1.878 mg/100 g), benzoic acid (1.643 mg/100 g), chlorogenic acid (1.567 mg/100 g), gallic acid (1.46 mg/100 g), hesperidin (0.889 mg/100 g), quercetin (0.737 mg/100 g), syringic acid (0.498 mg/100 g), *p*-coumaric acid (0.209 mg/100 g), and ellagic acid (0.105 mg/100 g). A similar study was conducted by Shekhar et al. [4] on bioactive components of garlic leaves. The results showed that concentration of polyphenols were aligns with the current findings.. The retention time (min) for the detected of gallic acid in the garlic sprout extract was revelated with previous study.. Fernandes et al. [42] used the HPLC-UV method for the determination of gallic acid in *Schinopsis brasiliensis* and observed a retention time of 8.5 min, which is close to the current study findings.

## 4 Conclusion

In this study, optimization was performed by applying response surface methodology for ultrasound extraction of *Allium sativum* sprout extracts. The present work was a contribution to expand the understanding of secondary metabolite extraction strategies from valuable garlic plant. The proximate results ensured the high nutritional composition of garlic sprouts, whereas microbial analysis and color measurement measured the safety concerns. However, chromatographic analysis ensured the characterization of polyphenols in garlic sprout extract. Garlic sprouts can be introduced to the food sector as a spice due to their good sources of bioactive compounds. Furthermore, sprouts powder can be used as a value additive material to enhance the shelf stability of different food products. In the future, further studies should be needed to aware peoples of the benefits of garlic sprouts. Moreover, sprout powder can be introduced into the pharmacological industry by investigating its nutritional and pharmacological potential.

## 5 Funding acquisition

The publication of this article was funded by the Open Access Fund of Leibniz Universität Hannover.

## CRediT authorship contribution statement

**Waseem Khalid:** Writing – review & editing, Writing – original draft, Software, Investigation, Formal analysis, Data curation. **Imed E. Benmebarek:** Writing – review & editing, Visualization, Validation, Methodology, Formal analysis. **Kanza Saeed:** Writing – review & editing, Visualization, Validation, Resources, Formal analysis. **Hyrije Koraqi:** Writing – review & editing, Visualization, Validation, Resources, Formal analysis. **Andres Moreno:** Writing – review & editing, Writing – original draft, Visualization, Validation, Supervision, Project administration, Methodology, Investigation, Formal analysis, Data curation, Conceptualization. **Robert Mugabi:** Writing – review & editing, Software, Data curation. **Gulzar Ahmad Nayik:** Writing – review & editing, Visualization, Resources, Data curation. **Tuba Esatbeyoglu:** Writing – review & editing, Supervision, Resources, Methodology, Funding acquisition, Data curation, Conceptualization.

## Data availability

Data will be made available on request.

## Declaration of competing interest

The authors declare that they have no known competing financial interests or personal relationships that could have appeared to influence the work reported in this paper.
